# Attitudes and behaviours on driving under the influence of drugs: a multigroup analysis of non-drug users and people who use methamphetamine

**DOI:** 10.1186/s12954-026-01400-6

**Published:** 2026-01-29

**Authors:** Aaron Mackay, Luke A. Downey, Shalini Arunogiri, Rowan P. Ogeil, Amie C. Hayley

**Affiliations:** 1 Centre for Mental Health and Brain Sciences, School of Health Sciences, Swinburne University of Technology, Hawthorn, VIC 3122 Australia; 2 Institute for Breathing and Sleep, Austin Health, Melbourne, Australia; 3 Monash Addiction Research Centre, Eastern Health Clinical School, Faculty of Medicine, Nursing and Health Sciences, Monash University, Melbourne, Australia; 4 Turning Point, Eastern Health, Richmond, VIC 3121 Australia; 5 International Council for Alcohol, Drugs and Traffic Safety (ICADTS), https://www.icadtsinternational.com/

**Keywords:** Methamphetamine, Attitude, Behaviour, Driving, Predictor, Risky

## Abstract

**Background:**

Stimulant-affected drivers are overrepresented in global road trauma statistics, however, studies to date have not accurately defined how drug consumption contributes to increased risk of road trauma. This study examined whether attitudes toward drug driving predicts dangerous driving behaviour among people who currently use methamphetamine, and whether this differs to individuals with no history of any drug use.

**Method:**

Three attitude factors (favourable attitudes toward risks, unfavourable attitudes toward sanctions, and favourable peer attitudes) were explored using an adapted version of attitudes towards drug driving scale and dangerous driving was measured using the Dula Dangerous Driving Index.

**Results:**

A multigroup structural equation model indicated that individuals who use methamphetamine report more favourable attitudes toward drug driving compared to those who have never used drugs. Among people who use methamphetamine, a favourable attitude towards drug driving risks predicted higher dangerous driving behaviour scores, while more unfavourable attitudes toward sanctions for drug driving predicted lower scores. Among those with no history of substance use, favourable peer attitudes toward drug driving predicted dangerous driving behaviour.

**Conclusions:**

Attitudes towards drug-driving, and their relationship to dangerous driving behaviour differs between those who use methamphetamine and those who do not have a history of substance usage. Targeted campaigns aimed specifically at reducing methamphetamine-related road trauma should challenge general underlying beliefs and attitudes about drug driving, rather than simply the impact of potential sanctions or influence of peers.

**Supplementary Information:**

The online version contains supplementary material available at 10.1186/s12954-026-01400-6.

## Introduction

Stimulant-affected drivers are overrepresented in global road trauma statistics. Amphetamine-type stimulants, predominantly methamphetamine, account for close to half of all traffic-related fatalities worldwide due to driving under the influence of drugs [1]. In Australia, motor vehicle collisions account for over 10% of all unintentional deaths for people who regularly consume this drug [2]. Despite the extensive scale and impact of methamphetamine consumption on road safety, research has yet to accurately define how consumption of this substance contributes to increased risk of road trauma. Therefore, detailed exploration of relevant intra- and inter-personal factors among people who regularly consume these substances may provide useful insight into predictors of behaviours that may ostensibly increase the risk for traffic-related harm.

The increasing supply, distribution, and rates of community consumption of methamphetamine correlates well with traffic-related harm [3]. Between the years 2015–2020, Australian national consumption (kilograms per annum) of methamphetamine increased by 32% [4]. In this same time period, incident rates of road trauma-related ambulance attendances involving methamphetamine increased by 87% [5]. Methamphetamine is now one of the most frequently detected illicit drugs among Australian drivers that are injured and killed due to a road traffic crash [6–8]. Methamphetamine-affected drivers are 5–30 times more likely to be seriously injured or killed in collision relative to sober drivers [9] – a risk at a level equivalent to that seen for high-range alcohol intoxication between 0.8 and 1.2 g/L [10]. International data indicates that one-in-ten people who recreationally consume methamphetamine, and one-third of individuals with a diagnosed stimulant-use disorder also report driving while intoxicated, suggesting a high prevalence of risky driving in this specific cohort [11, 12]. Despite robust population-level data showing increased prevalence, relative risk, and impact of methamphetamine consumption of traffic-related harm, acute drug administration studies have thus far been unsuccessful in determining exactly how acute intoxication translates to an elevated crash risk.

A moderate acute dose (~ 30 mg oral) of methamphetamine does not reliably impair performance [13], and has even shown to selectively *improve* neurocognitive skills ostensibly related to driving [14]. Limited driving simulator studies examining acute methamphetamine intoxication similarly report either a lack of impairment [15], or note a protection against gradual performance deterioration during a monotonous driving task [16]. Only one study has thus far examined driving performance among people who regularly consume methamphetamine [17]. Relative to matched drug-free controls, those individuals exhibited greater speed, lane weaving, and reduced headway during driving, even in the absence of acute intoxication. Due to justified ethical and methodological constraints, acute clinical experimental research cannot reliably replicate patterns of consumption reflective of heavier recreational patterns of use, and thus the contribution of more naturalistic patterns of use on driving outcomes, and its contribution to an elevated risk for harm, remains unclear.

Beyond acute intoxication, several distinguishable interpersonal factors, including attitudes and beliefs towards driving under the influence of drugs, may indirectly, or directly influence this type of driving behaviour based on perceived risk, likelihood of punishment and other social or peer-related pressures. Where they are strongly held, cognitively accessible, and internally consistent, attitudes have been shown to strongly predict behaviour [18, 19]. In the context of road safety, attitudes may influence actions that can be reasonably expected to increase risk of traffic-related harm (such as driving while impaired). Early studies on alcohol-impaired driving showed that positive endorsement of “risk behaviour” (driving while under the influence of alcohol) significantly correlated with participation in that behaviour (drink-driving) [20]. This has also been reported for drug-impaired driving (not drug-specific), whereby personal and social acceptance of drug driving more generally predicts engagement in that activity [21]. Problematic methamphetamine use is a strong predictor of drug driving, which in turn is associated with engagement in strategies to avoid being detected (e.g. taking ‘back streets when driving home’) [22]; however, it remains unknown what, if any, underlying beliefs and attitudes drive this behaviour in this specific cohort. Australian police traffic enforcement operations intended to reduce impaired driving (e.g. random breath alcohol and/or drug testing) assumes an inverse relationship between perceived risk of punishment and likelihood of offending [23]. This deterrent based approach is somewhat ineffectual when drivers actively disregard the risk of punishment and seek to circumvent it. Thus, it is important to explore relevant attitudes and behaviours related to drug driving among people who consume methamphetamine to most effectively inform and implement countermeasures to reduce traffic-related harm.

Australia has one of the highest global rates of methamphetamine consumption [24], and its use is implicated in a significant and growing proportion of national traffic-related harm. Discerning the effects of the methamphetamine on driving is challenging, not least due to the variations in the ways this drug affects driving, how these factors might feasibly interact with interpersonal factors, and the potential impact this has on overt traffic-related behaviours. Attitudes and beliefs towards driving under the influence of drugs may indirectly or directly influence driving behaviour based on perceived risk, and this is likely to differ among individuals who do or do not use substances. This study expands on previous work linking attitudes and behaviours towards drink-driving [25] by seeking to understand the relationship between attitudes towards drug driving and dangerous driving behaviour in individuals who self-report using methamphetamine, and assessing how this differs to a non-drug using cohort.

## Methods and materials

### Participants and procedure

Licenced Australian drivers aged 18–50 years who regularly consume methamphetamine and individuals who reported no history of any drug use were recruited through flyers distributed in the community, advertisements on social media platforms (Facebook and Twitter), and via targeted patient databases at Turning Point (Eastern Health); a clinical addiction treatment centre in metropolitan Melbourne. Participants completed a forced entry, branched logic anonymised online survey questionnaire that included a validated measure on self-reported dangerous driving behaviour and validated measure assessing attitudes toward drug driving. The survey was open for responses between 27th January 2021 and 27th September 2022.

Respondents who did not report any lifetime drug use (*Have you EVER used any drug other than alcohol ON YOUR OWN - that is*,* either WITHOUT a doctor’s prescription; in GREATER amounts*,* MORE OFTEN*,* or LONGER than prescribed; or for a reason other than a doctor said you should use them*”) were categorised as people with no drug use history. Those reporting any lifetime substance use underwent further screening to identify those with predominant and sustained methamphetamine use (at least once per month for at least 6 months during the period of heaviest consumption) and were categorised as methamphetamine users.

The study was approved by the Swinburne University of Technology Human Research Ethics Committee (SUHREC approval: 0215450-5806). Site-specific approval was obtained from the Eastern Health Human Research Ethics Committee to recruit participants. Survey completers had the option to voluntarily enter a draw for a chance to win a $100 gift voucher. Due to the survey’s sensitive nature, temporary email addresses were accepted, and contact details were scrambled and separated from the survey responses to ensure anonymity.

### Measures

#### Demographic factors

Demographic characteristics were collected at the start of the survey and included age (years), gender, highest education level achieved (university degree, high school/technical degree, did not finish university, did not finish high school), employment status (Student, Homemaker, Unemployed, Employed Part Time, Employed Full Time) and residential area (Urban/Inner-city, Rural/Suburban).

#### Alcohol and substance use characteristics

*Alcohol use*: Alcohol use was assessed using the Alcohol Use Disorder Identification Test-Consumption (AUDIT-C). The AUDIT-C is used as a measure to identify individuals who are prone to heavy drinking and who may be at risk of alcohol abuse [26]. The test consists of three questions that capture frequency of alcohol consumption. A composite score of ≥ 4 for men and ≥ 3 for women signifies that the participants drinking may be affecting their health and safety [27].

*Methamphetamine use*: The Severity of Dependence Scale (SDS) was used to assess current level of dependence on methamphetamine. The SDS is a five-item assessment that asks respondents to reflect on their drug use over the last 12 months. A cut-off scores of ≥ 5 is indicative of problematic amphetamine dependence [28].

Other characteristics relating to methamphetamine use that were captured included: frequency of methamphetamine use at peak (1 to 2 times per month, weekly or daily), last time using methamphetamine (years, months, weeks, or days), most common mode of methamphetamine administration (injection, smoking, snorting, oral) and any other illicit drug use (lifetime).

#### Attitudes toward drug driving

Attitudes toward drug driving were measured with an adapted drink-driving questionnaire developed by Davey et al. (2005) [25]^1^. The measure assesses 3 factors with 10 statements, with each item portraying a belief or attitude related to drug driving (e.g., *The dangers of drug driving are overrated*). Each item assesses the extent to which a participant agrees or disagrees with the statement on a 7-point Likert scale from “strongly disagree” to “strongly agree”. The three attitude factors include: attitude toward risk (where higher scores indicate a belief that the risks of drug driving are overrated); attitude toward sanctions (where higher scores indicate disproval of harsh sanctions for drug driving); and attitudes of peers (where higher scores indicate the approval of one’s peer group toward engaging in drug driving). A mean score for each factor is calculated as the mean of items after reverse scoring negatively worded items. A total mean score is calculated as the mean of all items and represents an overall approval or disproval toward drug driving, with higher scores indicating greater approval for drug driving (i.e., that drug driving is acceptable). The question “Everybody who takes drugs, drives under their influence once in a while” was omitted from analysis as it did not load onto a specific factor.

#### Dangerous driving behaviour

Dangerous Driving Behaviour was assessed using the Dula Dangerous Driving Index (DDDI); a self-report questionnaire that examines propensity to engage in dangerous driving behaviour based on self-reported statements [29]. The DDDI is made up of 28 statements that assess an individual’s propensity to engage in an aspect of dangerous driving behaviour (e.g., *I drive when I am angry or upset*). Items are rated on a 5-point Likert scale from ‘Never’ to ‘Always’ and the total score has been demonstrated to be a reliable and valid measure of real-world dangerous driving behaviour [30, 31].

### Statistical analyses

The psychometric properties of the drug-driving attitudes questionnaire were assessed using Cronbach’s alpha for internal consistency and confirmatory factor analysis for underlying factor structure. Multigroup structural equation modelling (SEM) was performed to assess whether the effect of drug driving attitudes (predictor variables) on dangerous driving behaviour as measured by the DDDI (outcome variable) varied between people who use methamphetamine and people who do not use drugs. To conduct the multigroup analysis, a chi-square difference test was first conducted between the free and constrained models to assess whether constraining parameters significantly degraded model fit.

Following this, each attitude-behaviour path was individually constrained and tested to identify which specific relationships differed between the two groups. Finally, the strength and direction of the significant paths were examined and interpreted within each group. All statistical analyses were conducted in R (version 4.2.3). Confirmatory factor analysis multigroup SEM; were performed using the Lavaan package [32].

### Sample characteristics

#### Demographics

Sample characteristics are presented in Table 1. Out of the 125 responses, 53 (62.26% Male) were for people who used methamphetamine and 72 (45.83% Male) were for people who did not use drugs. The mean age for people who used methamphetamine and people who did not use drugs was 30.77 (*SD* = 7, range = 20–50) and 28.31 (*SD* = 6.23, range = 18–50), respectively. Among individuals who used methamphetamine, 49 (92.45%) held a current and valid drivers’ licence, three had a revoked license and one had a former/lapsed license. All individuals who reported no lifetime drug use held a current and valid licence at the time of participation. Those who used methamphetamine were significantly less likely to have reported completing a university or high school/technical degree (52.83% vs. 81.94%) and were less likely to be in current employment compared to those who had no drug use history (67.92% vs. 87.5%, respectively). Those using methamphetamine had significantly higher levels of lifetime alcohol use (98.11% vs. 41.67%), current at-risk drinking behaviour (66.04% vs. 15.28%) and self-reported psychiatric diagnoses (any) (92.45% vs. 43.06%) compared to those with no drug use history (all *p <* .05).

#### Methamphetamine use

The majority (94.34%) of the sample of individuals who use methamphetamine met the criteria for dependence (i.e., they returned an SDS score of 5 or greater on the SDS). Considering lifetime use, 62.26% reported using methamphetamine either daily or weekly at peak usage, with 77.36% of these individuals reporting injecting the drug (20.75% reported smoking it and 1.89% reported using it orally). The average age at first use was 23.51 years (SD = 5.27).

##### Psychometric properties of attitudes survey

Attitudes toward risk and attitudes toward sanctions both demonstrated good internal consistency ($\alpha=.73$ and $\alpha=.88$ respectively). Peer attitudes demonstrated moderate internal consistency ($\alpha=.6)$. Factor loadings for each item can be seen in Table 2.

**Table 1:** Sample demographic characteristics

Characteristic	People who use Methamphetamine *N* = 53	People with no drug use history *N* = 72
Age (Years) Mean (SD)	30.77 (7.04)	28.31 (6.23)
Sex		
Male	33 (62.26%)	33 (45.83%)
Female	20 (37.74%)	37 (51.39%)
Prefer not to say	0 (0%)	2 (2.78%)
Education		
University Degree	8 (15.09%)	46 (63.89%)
Highschool/Technical Degree	20 (37.74%)	13 (18.06%)
Did not finish University	16 (30.19%)	13 (18.06%)
Did not finish High School	9 (16.98%)	0 (0%)
Alcohol Use (Ever)	52 (98.11%)	30 (41.67%)
AUDIT-C Score	5.02 (2.24)	1.53 (2.53)
Employment status		
Employed full time	21.00 (39.62%)	52.00 (72.22%)
Employed part time	15.00 (28.30%)	11.00 (15.28%)
Unemployed looking for work	10.00 (18.87%)	0.00 (0.00%)
Student	0.00 (0.00%)	7.00 (9.72%)
Homemaker	4.00 (7.55%)	1.00 (1.39%)
Unemployed not looking for work	3.00 (5.66%)	0.00 (0.00%)
Retired	0.00 (0.00%)	1.00 (1.39%)
License status		
Valid/current	49.00 (92.45%)	72.00 (100.00%)
Revoked	3.00 (5.66%)	0.00 (0.00%)
Former/lapsed	1.00 (1.89%)	0.00 (0.00%)
Psychiatric diagnosis	49.00 (92.45%)	31.00 (43.06%)

**Table 2:** Factor loadings and Cronbach’s $\alpha$ for each factor

Variables	Factor loadings
Attitudes towards risk	
The dangers of drug driving are overrated	0.6
The police aren’t tough enough on drug drivers	0.4
It’s OK to drug drive as long as you don’t get caught	0.76
It’s OK to drive after taking drugs as long as you’re not too high	0.87
Cronbach’s $\alpha$ = 0.73	
Attitudes towards sanctions	
My community needs stricter laws against drug driving	0.92
People who take drugs and drive should go to jail	0.9
People who take drugs and drive should lose their driver’s license	0.74
Cronbach’s $\alpha$ = 0.88	
Perceived peer attitude	
Most of my friends think it’s OK to take drugs and drive	0.76
My friends would think I was dumb if I drove after taking drugs	0.56
Cronbach’s $$\alpha$$ = 0.6

**Table 3:** Regression model for people who use methamphetamine with dangerous driving as dependent variable

Term	Estimate		95% CI		SE		Cohen’s f^2^		*p*
(Intercept)	59.83		40.93–78.72		9.40		-		< 0.001
Favourable attitudes toward risk	9.64		3.57–15.71		3.02		0.08		0.002
Unfavourable attitudes toward sanctions	-8.23		-13.08 - -3.39		2.41		0.10		0.001
Favourable peer attitudes	0.21		-4.65–5.07		2.42		0.00		0.933

**Table 4:** Regression model for people with no history of any drug use with dangerous driving as dependent variable

Term	Estimate	95% CI	SE	Cohen’s f^2^	*p*
(Intercept)	26.53	17.6–35.45	4.47	-	< 0.001
Favourable attitudes toward risk	0.49	-3.38–4.36	1.94	0.00	0.801
Unfavourable attitudes toward sanctions	2.62	-0.63–5.88	1.63	0.02	0.113
Favourable peer attitudes	7.51	4.12–10.9	1.70	0.12	< 0.001

#### Comparison of attitudes between groups

**Fig. 1:**
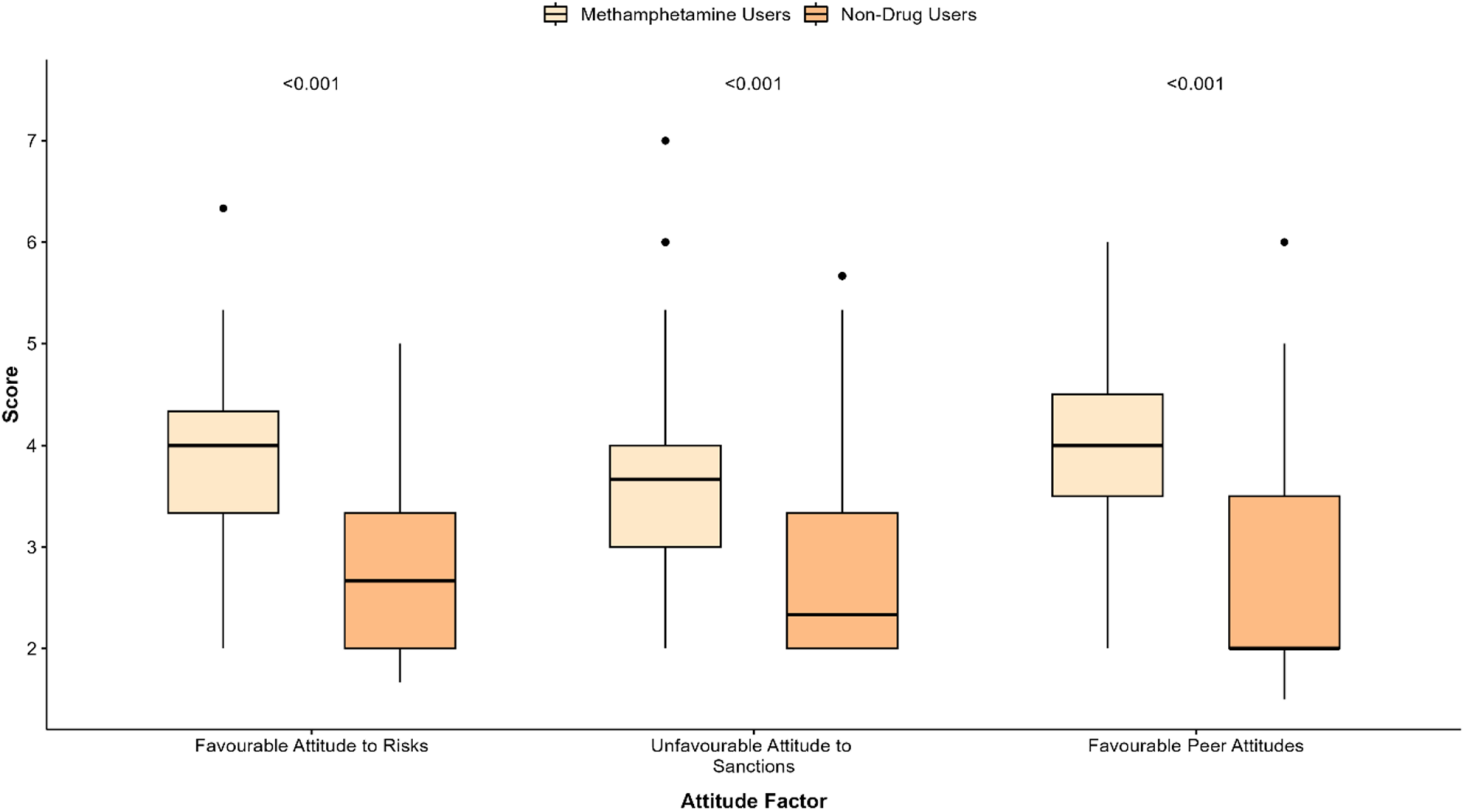
Boxplots of drug driving attitudes factors with comparison between drug using group

The mean overall attitude toward drug driving was significantly greater (i.e. more favourable attitudes toward drug driving) for people who use methamphetamine [M = 3.98 (95% CI = 2.77–4.20)], compared to people with no reported drug use [M = 2.78 (95% CI = 2.60–2.97) (t_(114)_ = -8.58, *p* < .001). Compared to people who do not use drugs, those who used methamphetamine demonstrated significantly higher scores across all three attitude factors (Fig. 1), and reported higher levels of self-reported dangerous driving behaviour [M = 66, SD = 15 vs. M = 55, SD = 14; t_(110)_ = -4.38, *p* < .001].

#### Multigroup analysis

##### Invariance test

The results of the overall invariance test showed a significant difference between the free and constrained model [${\chi}^{2}diff=26.22,p<.001$].

##### Constraining individual paths

Constraining *attitudes toward risk* between the free and constrained model indicated a significant difference in the path coefficient for risk [${\chi}^{2}diff=6.74,p<.01]$. Constraining *attitudes toward sanctions* revealed a significant difference in the path coefficient between the free model and the model constraining sanctions [${\chi}^{2}diff=13.67,p<.001]$. Constraining *peer attitudes* resulted in a significant difference in the path coefficient between the free model and model constraining peer attitudes [${\chi}^{2}diff=6.35,p<.05]$.

##### Paths within each group

*People who use Methamphetamine*

Among the group of people who use methamphetamine, the regression model explained 22% of the variance in self-reported engagement in dangerous driving behaviour (R^2^ = 0.22, adjusted R^2^ = 0.17). Within this model, favourable attitudes toward risk had a significant, positive relationship with higher scores on the DDDI ($b=9.64,\beta=0.60,p<.01$) when controlling for attitudes toward sanction and peer attitudes. Favourable attitudes toward sanctions within this group had a significant, negative relationship with dangerous driving ($b=-8.23,\beta=-0.62,p<.01)$ when controlling for attitudes toward risk and peer attitudes. Favourable peer attitudes toward drug driving had a non-significant, positive relationship with dangerous driving $(b=0.21,\beta=0.01,p=.93)$ (Table 3).

*People with no history of any drug use*.

Among the group of people with no history of any drug use, the regression model explained 52% of the variance in self-reported engagement in dangerous driving behaviour (R^2^ = 0.52, adjusted R^2^ = 0.50). Within this model, favourable attitudes toward risk had a non-significant, positive association with dangerous driving when controlling for attitudes toward sanctions and peer attitude $(b=0.49,\beta=0.03,p=.80)$. Favourable attitudes toward sanctions had a non-significant, positive association with dangerous driving $(b=2.62,\beta=0.21,p=.11)$. Favourable peer attitudes toward drug driving had a significant, positive relationship with dangerous driving $(b=7.51,\beta=0.54,p<.001)$ (Table 4).

#### Model fit

All baseline models were just identified with zero degrees of freedom (${\chi}^{2}$= 0 for all models). The model for people who use methamphetamine yielded an AIC of 430.59, while the model for people with no drug use history yielded an AIC of 542.38. When both groups were modelled simultaneously in the multi-group analysis, the combined AIC was 976.97.

## Discussion

The study explored whether the relationship between attitudes toward drug driving and dangerous driving behaviour differed between people who use methamphetamine and people with no history of any drug use. We found that among people who consume methamphetamine, a more favourable attitude toward the risks of drug driving predicts higher engagement in dangerous driving behaviour ($\beta=0.60)$, while more unfavourable attitudes toward sanctions for drug driving (i.e., beliefs that the laws and punishment for drug driving are too harsh) predict lower engagement ($\beta=-0.62)$, despite higher overall disapproval of harsh sanctions. These moderate-to-large effect sizes suggest that both risk perception and attitudes toward punishment play substantial roles in driving behaviour within this population. Among individuals with no history of any drug use, peer attitudes were significantly and positively associated with dangerous driving behaviour ($\beta=0.54)$, while attitudes toward risk and attitudes toward sanctions were not. Targeted campaigns aimed specifically at reducing road trauma among people who regularly use methamphetamine should therefore more clearly seek to highlight risks and dangers associated with such behaviour, rather than simply focussing on incurred sanctions. Conversely, campaigns intended to frame dangerous driving as socially unacceptable, thereby challenging peer perceptions and negative influence, may help prevent engagement in these behaviours among drivers who do not consume drugs.

Australian drivers who regularly consume methamphetamine report more favourable attitudes toward drug driving compared to those who have no history of any drug use. They also express greater disapproval of harsh sanctions for drug driving. Among these drivers, favourable attitudes towards risk predicted greater engagement in dangerous driving, suggesting that a permissive attitude towards risks and disregard for the consequences of impaired driving may influence participation in this behaviour, irrespective of perceptions of its potential social (un)desirability. These findings may, in part, help explain the somewhat negligible impact that enforcement alone has had on national drug-driving recidivism rates, which have largely remained unchanged in this cohort despite increased visibility [23]. Circumstantially, individuals who regularly use methamphetamine may be more inclined to drive dangerously due to direct and peripheral factors related to patterns of use. Specifically, convenience of driving after sourcing/using methamphetamine, and lack of suitable alternative transportation methods may contribute to the decision to drug-drive [33]. Between 30 and 40% of people who regularly consume methamphetamine report purposefully driving while intoxicated [34], and they are also more than three-times more likely to drive in a dangerous manner while under the influence [11]. Risky driving (such as speeding or evasive driving to avoid detection) is frequently self-reported by this group [35] and this behaviour has been deliberately linked with acute drug consumption [36]. Interestingly, methamphetamine is typically perceived to be less impairing than other illicit substances [37, 38], and is judged to have negligible detrimental (or even beneficial) effects on driving by those who regularly consume it [34]. Thus, it appears that these drivers underestimate (or disregard) the risk that their methamphetamine use poses to traffic-safety [39, 40], and voluntarily engage in reckless driving practices that can increase harm [33]. There is a rich literature around cognitive impacts of acute and chronic methamphetamine use that suggests an endearing negative impact on cognition, specifically on decision making in relation to immediate and future risk/reward [41]. Consequently, purely enforcement-based countermeasures are unlikely to impart meaningful behavioural change without adequate psychosocial support or intervention [23, 33]. In seeking to reduce harm, it may therefore be more effective to focus on behaviour change programs that are integrated with drug treatment programmes and services that target these mechanisms (e.g., goal management training). Specifically, this approach should seek to challenge general risk estimation, sensitivity to punishment and explore the role of reward or anger in relation to driving to better implement longer-term strategies to support deterrence.

A drivers’ social group can influence behaviours by encouraging (or dissuading) risk taking [42]. Among this sample of drivers who have no history of any drug use, peer attitudes significantly predicted dangerous driving behaviour after controlling for attitudes toward risk and sanctions. Specifically, as peer attitudes become more favourable to drug driving, propensity to drive dangerously increased, suggesting that individuals with peer groups who hold more approving views on drug driving are more likely to drive dangerously than people whose peer groups disapprove of such behaviour. Peer influence is critical in shaping both formal and informal models of behaviours and can significantly influence road safety. Antisocial peer influence, lack of belief about the risks of dangerous/risky driving and certain demographic factors predict engagement in dangerous driving practices and incidence of serious traffic collisions (damage, casualties), particularly among younger (18–25 years) or more inexperienced drivers (such as probationary drivers) [43, 44]. The present cohort comprised fully licenced young adult drivers (mean age 28 years), as such, the pervasiveness of negative social influence in shaping behaviours exist even beyond the younger-inexperienced typically ‘vulnerable’ period. Peer-led behavioural change and driver training programmes that seek to challenge entrenched attitudes and address anti-social road user behaviour can impart positive change by reducing incidence of dangerous driving [45]. Thus, targeted, prosocial early-intervention educative strategies may similarly help to reduce harm by safeguarding against negative peer influence, particularly concerning drug-impaired driving and its potential to peripherally influence engagement in more risky driving practices. Neither attitude toward risk nor attitudes toward sanctions significantly influenced propensity to engage in dangerous driving among this sample, suggesting that existing safety messaging and traffic sanctions are effective in influencing road-rule compliance.

In interpreting these results, several important limitations are noted. To our knowledge, this is the largest and most targeted investigation of attitudes towards risky driving among Australian drivers who use methamphetamine. Our inclusion criteria were purposefully strict, and we implemented numerous checks to vet individuals who did not report predominant use of methamphetamine. This approach, whilst effective in strengthening the quality of the acquired responses and associated data, likely contributed to the low number of respondents. Given this purposeful specificity of the study cohort, we are unable to comment on the magnitude of effect and the strength of attitude paths for people who principally consume substances other than methamphetamine, or whether these associations may differ in regions or jurisdictions with differing legislative or enforcement frameworks. Thus, replication studies should seek to recruit a broader cohort of licenced individuals who consume drugs and consider including a region-specific comparator cohort. Internal consistency and factor loadings of the adapted drink-driving questionnaire were comparable to the original drink driving measure [46] suggesting that the adapted survey used here is reliable and fit for purpose.

## Conclusion

Drug driving attitudes, and their relationship to dangerous driving behaviour differs between those who use methamphetamine and those who with no history of any drug use, indicating that methamphetamine use status determines the unique relationship between attitudes toward drug driving (including risk, sanctions, and peer attitudes) and engagement in dangerous driving behaviour. Targeted road safety messages are thus encouraged to assist in developing and enacting effective countermeasures relevant to each road user cohort.

## Supplementary Information

Below is the link to the electronic supplementary material.

Supplementary Material 1

Supplementary Material 2

Supplementary Material 3

Supplementary Material 4

Supplementary Material 5

## Data Availability

The datasets used and/or analysed during the current study are available from the corresponding author on reasonable request.

## Footnotes

1 The original questionnaire was designed to assess attitudes toward drink driving [46]. The adapted measure reduced the number of items and factors from the original questionnaire and replaced *drink driving* with *drug driving*.
