# Supplementary material for: Attitudes and behaviours on driving under the influence of drugs: a multigroup analysis of non-drug users and people who use methamphetamine
Source: Harm Reduct J. 2026 Jan 29;23:40. doi: 10.1186/s12954-026-01400-6 (PMC12922437; doi:10.1186/s12954-026-01400-6)
Supplement: Supplementary file 2 — Supplementary Material 2 [file 12954_2026_1400_MOESM2_ESM.docx]

**Table A1. Questionnaire Development**

| **Factor** | **Baum et al. (1998)** | **Davey et al. (2005)** | **Present Study** |
| --- | --- | --- | --- |
| **Risk** | Dangers of drink driving are overrated | The dangers of drink driving are overrated | The dangers of drug driving are overrated |
|  | Police hassle drink drivers | The police aren't tough enough on drink drivers | The police aren’t tough enough on drug drivers* |
|  | It is ok to drink and drive as long as you are not caught | It's OK to drink and drive as long as you don't get caught | It’s OK to drug drive as long as you don’t get caught |
|  | It is ok to drink and drive as long as you are not drunk | It's OK to drive after drinking as long as you're not too drunk | It’s OK to drive after taking drugs as long as you’re not too high |
| **Behaviour** | I think it is okay to drive after drinking x drinks | - | - |
|  | I won't drive after drinking x drinks | - | - |
| **Sanctions** | My community needs stricter laws against drunk driving | My community needs stricter laws against drink driving | My community needs stricter laws against drug driving* |
|  | People who drink and drive should go to jail | People who drink and drive should go to jail | People who take drugs and drive should go to jail* |
|  | People who drink and drive should lose their drivers licence | People who drink and drive should lose their driver's license | People who take drugs and drive should lose their license* |
| **Peer** | Most of my friends think it's okay to drink and drive | Most of my friends think it's OK to drink and drive | Most of my friends think its OK to take drugs and drive |
|  | My friends would think I was really stupid if I drove after drinking | My friends would think I was dumb if I drove after drinking alcohol | My friends would think I was dumb if I drove after taking drugs* |
| **Not Loaded** | I will get picked up if I drove after drinking | - | - |
|  | Everybody drinks and drives once in a while | Everybody who drinks alcohol, drives under the influence once in a while | Everybody who takes drugs, drives under their influence once in a while |

**Reverse Scoring Items*
