# Supplementary material for: Attitudes and behaviours on driving under the influence of drugs: a multigroup analysis of non-drug users and people who use methamphetamine
Source: Harm Reduct J. 2026 Jan 29;23:40. doi: 10.1186/s12954-026-01400-6 (PMC12922437; doi:10.1186/s12954-026-01400-6)
Supplement: Supplementary file 4 — Supplementary Material 4 [file 12954_2026_1400_MOESM4_ESM.docx]

**Table A3. SEM Model Fit for people who use methamphetamine**

| **Variable** | **Value** |
| --- | --- |
| Model Parameters | 4 |
| # Observations | 53 |
| **User Model** |  |
| 𝜒2 | 0 |
| df | 0 |
| 𝑝 | NA |
| **Baseline Model** |  |
| chisq | 13.01 |
| df | 3 |
| Pvalue | <.01 |
| **Fit Measures**  **User Model vs Baseline** |  |
| Comparative Fit Index (CFI) | 1.0 |
| Tucker-Lewis Index (TLI) | 1.0 |
| **Loglikelihood and Information Criteria** |  |
| Loglikelihood user model (H0) | -211.30 |
| Loglikelihood unrestricted model (H1) | -211.30 |
| Akaike Information Criterion (AIC) | 430.59 |
| Bayesian Information Criterion (BIC) | 438.47 |
| Sample-size Adjusted Bayesian Information Criterion (SABIC) | 425.91 |
| **Root Mean Square Error of Approximation** **(RMSEA)** |  |
| RMSEA | 0.0 |
| RMSEA (lower) | 0.0 |
| RMSEA (Upper) | 0.0 |
| p-value | NA |
| **Standardised Root Mean Square Residual** **(SRMSR)** |  |
| SRMSR | 0.0 |
