# Supplementary material for: Attitudes and behaviours on driving under the influence of drugs: a multigroup analysis of non-drug users and people who use methamphetamine
Source: Harm Reduct J. 2026 Jan 29;23:40. doi: 10.1186/s12954-026-01400-6 (PMC12922437; doi:10.1186/s12954-026-01400-6)
Supplement: Supplementary file 5 — Supplementary Material 5 [file 12954_2026_1400_MOESM5_ESM.docx]

| Regression Model (MA Users) | | | | | | | |
| --- | --- | --- | --- | --- | --- | --- | --- |
| **Term** | **Estimate** | **Std. Beta** | **95% Ci** | **Se** | **Partial** | **F2** | **P** |
| (Intercept) | 59.83 |  | 40.93 - 78.72 | 9.4 | - | - | <0.001 |
| duid.att.risk | 9.64 | 0.603 | 3.57 - 15.71 | 3.02 | 0.41 | 0.08 | 0.002 |
| duid.att.sanction | -8.23 | -0.615 | -9.69 | 2.41 | -0.44 | 0.1 | 0.001 |
| duid.att.peer | 0.21 | 0.126 | -9.72 | 2.42 | 0.01 | 0 | 0.933 |
|  |  |  |  |  |  |  |  |
| R2 = 0.218 |  |  |  |  |  |  |  |
| Adjusted R2 = 0.170 |  |  |  |  |  |  |  |
|  |  |  |  |  |  |  |  |
|  |  |  |  |  |  |  |  |
|  |  |  |  |  |  |  |  |
|  |  |  |  |  |  |  |  |
|  |  |  |  |  |  |  |  |
| Regression (Non - MA Users) | | | | | | | |
| **Term** | **Estimate** | **Std. Beta** | **95% Ci** | **Se** | **Partial** | **F2** | **P** |
| (Intercept) | 26.53 |  | 17.6 - 35.45 | 4.47 | - | - | <0.001 |
| duid.att.risk | 0.49 | 0.027 | -7.74 | 1.94 | 0.03 | 0 | 0.801 |
| duid.att.sanction | 2.62 | 0.209 | -6.51 | 1.63 | 0.19 | 0.02 | 0.113 |
| duid.att.peer | 7.51 | 0.541 | 4.12 - 10.9 | 1.7 | 0.47 | 0.12 | <0.001 |
| R2 = 0.522 |  |  |  |  |  |  |  |
| Adjusted R2 = 0.501 |  |  |  |  |  |  |  |
